# Analysis of fouling and breakthrough of process related impurities during depth filtration using confocal microscopy

**DOI:** 10.1002/btpr.3233

**Published:** 2022-01-26

**Authors:** Maria Parau, Thomas F. Johnson, James Pullen, Daniel G. Bracewell

**Affiliations:** ^1^ Department of Biochemical Engineering University College London London UK; ^2^ FUJIFILM Diosynth Biotechnologies Billingham UK

**Keywords:** bioprocessing, CHO cell culture, depth filter, downstream processing, harvest

## Abstract

Titer improvement has driven process intensification in mAb manufacture. However, this has come with the drawback of high cell densities and associated process related impurities such as cell debris, host cell protein (HCP), and DNA. This affects the capacity of depth filters and can lead to carryover of impurities to protein A chromatography leading to early resin fouling. New depth filter materials provide the opportunity to remove more process related impurities at this early stage in the process. Hence, there is a need to understand the mechanism of impurity removal within these filters. In this work, the secondary depth filter Millistak+ X0HC (cellulose and diatomaceous earth) is compared with the X0SP (synthetic), by examining the breakthrough of DNA and HCP. Additionally, a novel method was developed to image the location of key impurities within the depth filter structure under a confocal microscope. Flux, tested at 75, 100, and 250 LMH was found to affect the maximal throughput based on the max pressure of 30 psi, but no significant changes were seen in the HCP and DNA breakthrough. However, a drop in cell culture viability, from 87% to 37%, lead to the DNA breakthrough at 10% decreasing from 81 to 55 L/m^2^ for X0HC and from 105 to 47 L/m^2^ for X0SP. The HCP breakthrough was not affected by cell culture viability or filter type. The X0SP filter has a 30%–50% higher max throughput depending on viability, which can be explained by the confocal imaging where the debris and DNA are distributed differently in the layers of the filter pods, with more of the second tighter layer being utilized in the X0SP.

## 1 INTRODUCTION

In recent years, mAb titers have increased beyond 5 g/L in 14‐day CHO fed‐batch cultures, enabling reduction of bioreactor sizes to maintain levels of demand.^1^ The disadvantage of high titers is the associated increased cell density, often exceeding 20 million cells/ml.^2^ This poses a significant problem for primary recovery to remove more biomass. Process related impurities are also of concern as higher cell density will naturally release more HCPs. HCP species that co‐elute with mAb are also more difficult to remove with Protein A at high cell density.^3^ It has also been shown that the choice of secondary clarification steps directly affected the HCP profile in ProA eluent.^4^ There are concerns that ProA resins will foul quicker when exposed to high impurity load, leading to increased costs and potential problems during manufacture.

Depth filtration is a popular choice for primary recovery of CHO cell culture due to the reduced shear rates compared to centrifugation and especially its suitability for single‐use facilities. 2000L single‐use bioreactors are a popular choice with some companies choosing to run several in tandem.^1^ At this scale, depth filtration is the typical choice for primary recovery. It has the advantage of being easier to operate compared to a centrifuge, highly flexible and requires less capital investment as well as being fully disposable.^5^

A filtration train is used at harvest to make the process more efficient. It is typically comprised of three filter trains, a coarse depth filter to remove the cells, a finer depth filter to remove colloidal matter and a 0.2 μm bioburden filter.^6^, ^7^ Each depth filter is also made up of several layers of media with different nominal pore sizes. The first layer is a coarse media that traps larger particles, followed by a tighter depth filter that clears colloidal and sub‐micron particles. This arrangement of the media layers helps to increase the holding capacity of the filter at fixed loading.

Depth filters have been traditionally made from cellulose fiber backbone, a porous filter‐aid such as diatomaceous earth and an ionic charged resin binder.^7^ Recently manufactures have begun moving away from natural materials to ensure better consistency during filter manufacture^8^, ^9^ and future supply as DE is a finite resource. In comparison to membranes used in bioprocessing that are specified with a single nominal retention rating, depth filters are often given a range instead given their wide pore structure.^10^

In addition to removal of material based on size exclusion, depth filters have been shown to remove soluble impurities by adsorption through hydrophobic, ionic, and other interactions.^7^ They have been used to remove endotoxin from water,^11^ and DNA from cell culture supernatant.^12^, ^13^ It has been shown that positively charged depth filters can reduce HCP and reduce the turbidity of Protein A chromatography eluate.^14^, ^15^

However, depth filters are struggling to cope with the high cell density and current set‐ups are reaching maximum holding capacity for solids removal. Holding capacity expansion is only available by an increase in filter number and facility footprint. There is also an increased interest to capture more soluble impurities before the chromatography stage. Therefore, organizations are looking to make their current processes more efficient, rather than expanding manufacturing space with cost being the major driver.^1^ Compared to other unit operations, such as chromatography, depth filtration has not been characterized in as much detail. Hence, there is a need to better understand the separation mechanisms.

The objective of this study is to examine the performance of Millistak+ depth filters, in order to develop an improved understanding of the mechanism of DNA and HCP removal when challenged with a high cell density CHO cell culture. This work investigates the effect of flux, cell culture viability and secondary depth filter materials during the harvest of CHO cell culture. Additionally, a novel confocal laser scanning microscopy (CLSM) method was developed to identify the distribution of foulant within the depth filter structure after they are utilized in the harvest experiments.

## 2 MATERIAL AND METHODS

### 2.1 Cell culture conditions

A mAb feedstock produced in Chinese Hamster Ovary (CHO) cells was provided by FUJIFILM Diosynth Biotechnologies utilizing their Apollo X™ platform. Several batches of the material were produced in 2 L shake flasks with cell density approx. 30 million cells/ml and harvested on varying days to achieve a difference in viabilities.

### 2.2 Depth filtration

**FIGURE 1:**
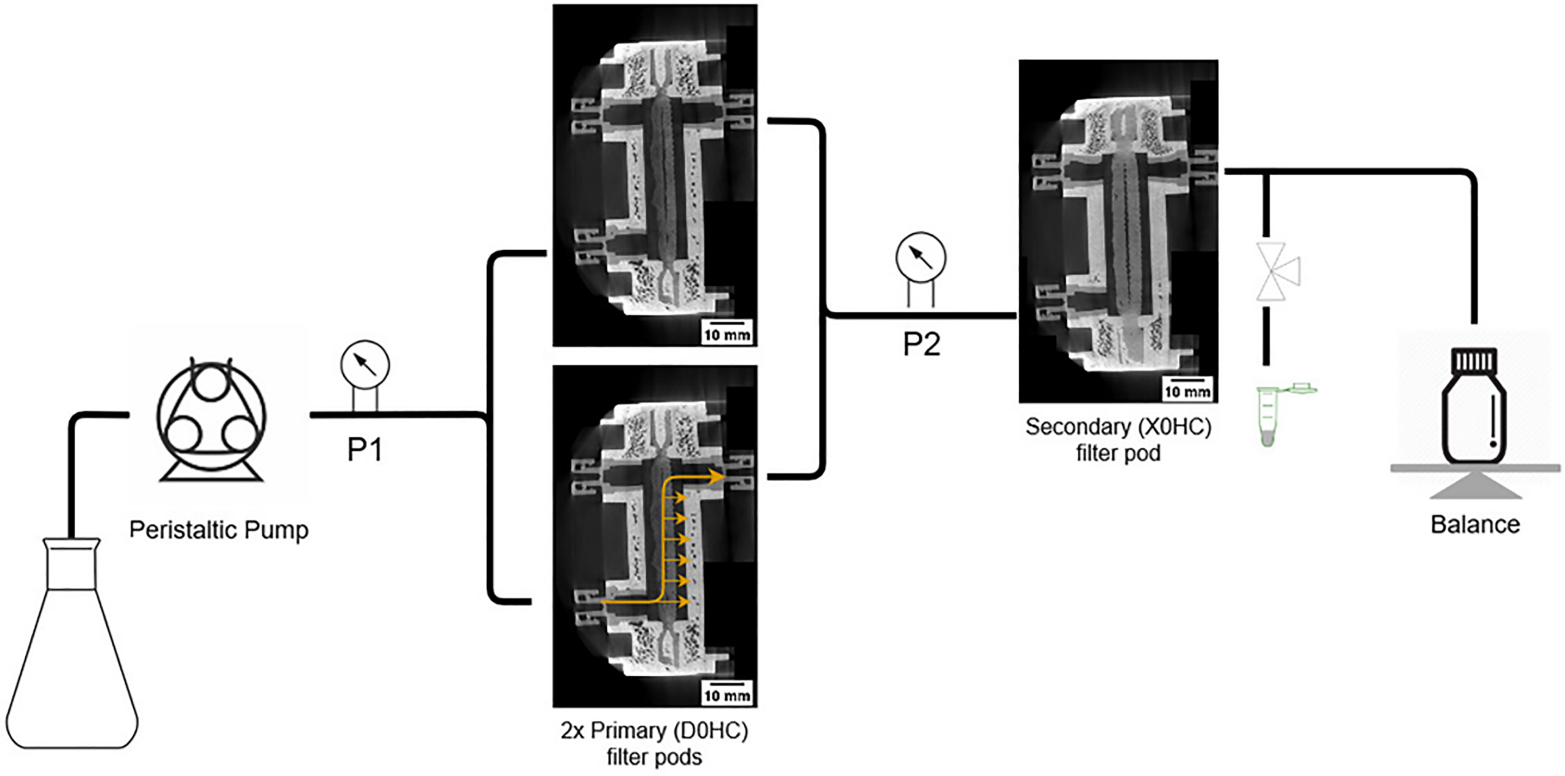
Schematic of depth filtration scale down model, with CT X‐ray cross‐section images of Millipore Millistak+ μPods D0HC (primary) and X0HC (secondary). The yellow arrow indicates the direction of flow in the filter pods

The filtration experiments were carried out in two parts. Figure 1 shows the scale down process used to mimic a 2:1 primary to secondary filters used at the manufacturing scale. Two Millipore Millistak+ D0HC 23 cm^2^ pods were operated in parallel and both feeding into one X0HC 23 cm^2^ pod. All experiments were operated at a constant flux of 150 LMH unless stated otherwise.

In the second part of the depth filtration experiments, the primary filter was scaled up to a 270 cm^2^ D0HC filter pod. The intermediate filtrate was collected and then directed through either X0HC 23 cm^2^ or X0SP 23 cm^2^ filter pod. This setup was repeated with four batches of CHO cell culture at different viability.

### 2.3 DNA, HCP, and mAb quantification

DNA concentration was measured using the QuantIT™ PicoGreen® dsDNA Kit (ThermoFisher Scientific), as per the manufacturer's protocol. Total protein concentration was measured using Pierce™ Rapid Gold BCA Protein Assay Kit (ThermoFisher Scientific), as per the manufacturer's protocol.

IgG quantification was performed by Protein A HPLC. A POROS(R) A20 column (Thermo Scientific) was connected to an Agilent 1200 Series HPLC (Agilent, Santa Clara, CA.). The sample injection volume was 100 μl. Equilibration was performed with 20 mM sodium phosphate, 300 nM NaCl pH 7.2. Elution was performed with 20 mM sodium phosphate, 300 nM NaCl pH 2.5. The flow rate was 1.5 ml/min. Concentration was calculated using an in‐house IgG1 standard.

HCP concentration was calculated as per equation below:

$$\left[\mathrm{HCP}\right]=\left[\text{Total protein}\right]-\left[\mathrm{IgG}\right].$$

### 2.4 X‐ray computed tomography

Imaging of depth filter pods was performed using a Nikon XT H 225 (Tring, UK) industrial scanner (accessed at the Electrochemical Innovation Laboratory, UCL). Settings included a 100 keV primary accelerating voltage using a silver target, 1 s exposure time and four frames averaged over 3142 projections with “Reduce ring artefacts” activated.^16^ Reconstruction was performed using Nikon X‐Tek software.

### 2.5 Confocal laser scanning microscopy

Filters were removed from the pods after use in the experiments described above and a 10 mm disc was cut from the middle of each filter. Filter discs were fixed in 4% PFA overnight and then frozen at −20°C. Slices were cut using a cryostat and transferred to a 24‐well plate with PBS buffer and 10 μl PicoGreen® (ThermoFisher Scientific) and 10 μl Nile Red (ThermoFisher Scientific) fluorescent dyes. The samples were placed on a shaker, protected from light, for 30 min. The samples were then washed with PBS for 10 min. Samples were transferred to a concave microscope slide with two drops of Vectashield. The cover glasses were sealed with clear nail polish and slides stored at 4–8°C and protected from light.

A Leica TCS SPE inverted CLSM (Leica Microsystems) was used for imaging the filter samples. Microscope settings were kept constant for all samples: magnification ×10, excitation wavelength at 488 and 561 nm, respectively, for PicoGreen and Nile Red dyes. Emission was captured at 500–550 nm and 600–750 nm, respectively. The automated platform was used to capture images in the *x*–*y* direction and these were combined in LAS‐X software.

Image analysis was performed using the software ImageJ. From each image, three areas were selected for sampling and the average integrated density was calculated, which is defined as the total sum of pixels in the sample.

### 2.6 Scanning electron microscopy

Images of the depth filters were acquired with a Zeiss Supra 55 VP electron microscope. The voltage was set at 5 kV. Each filter layer was imaged from the inlet side at 250, 500, and 1000× magnification.

## 3 RESULTS AND DISCUSSION

### 3.1 Scale‐down model and depth filter composition

A scale‐down depth filtration train was created to mimic the 2:1 ratio of primary to secondary filters. This was carried out using the Millipore Millistak+ HC series, which is composed of a cellulose‐based backbone and diatomaceous (DE) filler. Figure 1 shows the flowsheet of the scale down model used. Briefly, a peristaltic pump is used to feed the cell culture directly into two primary D0HC filters, which feed into one secondary X0HC system. A valve is used to sample the filtrate to create the DNA and HCP breakthrough curves.

**FIGURE 2:**
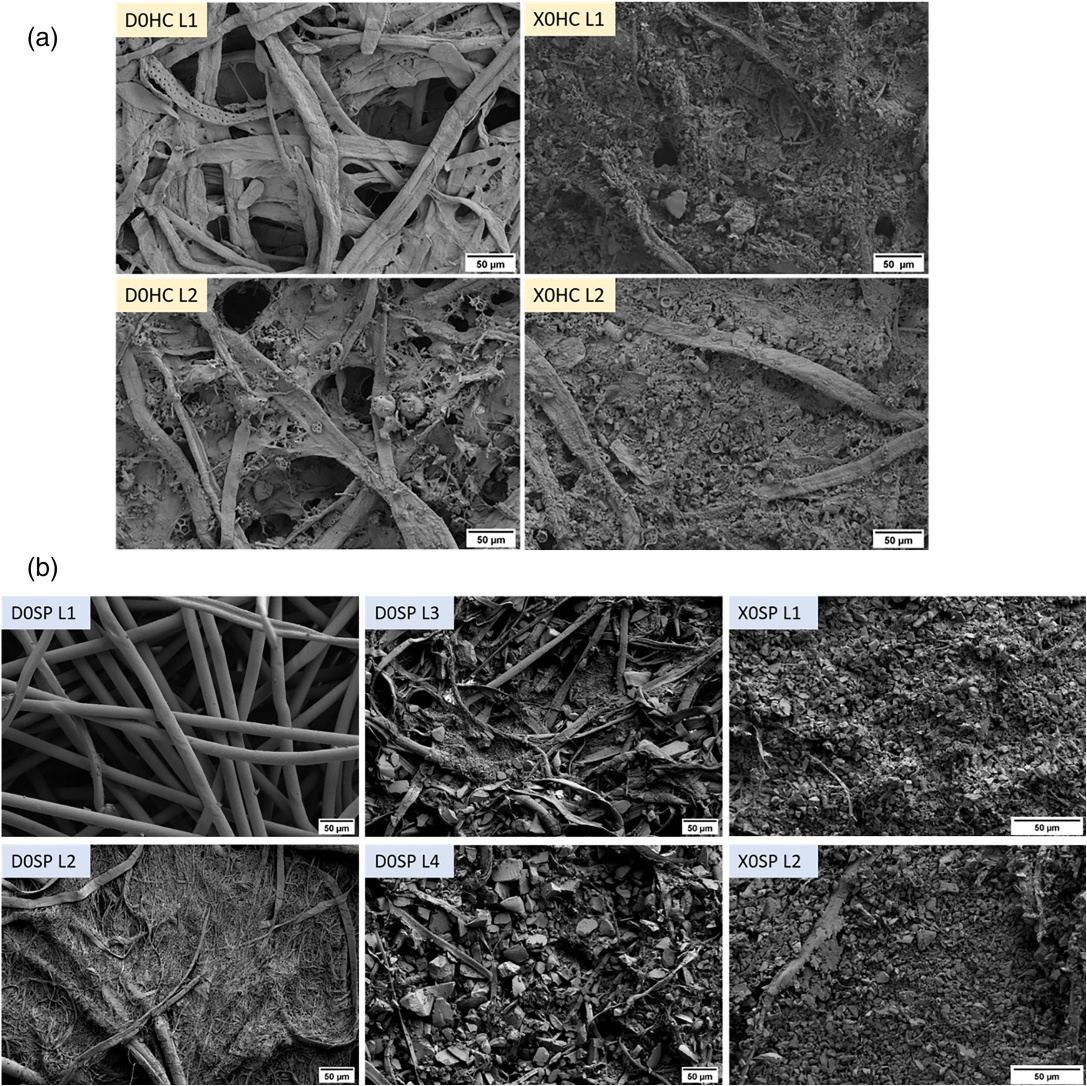
(a) Scanning electron microscopy images of the cellulose and DE‐based Millistak+ D0HC (primary) and X0HC (secondary). (b) Scanning electron microscopy images of the synthetic based Millistak+ D0SP (primary) and X0SP (secondary)

X‐ray computed tomography images were taken of the unused D0HC and X0HC filters. It can be seen in Figure 1 that both pods are composed of two layers. This multi‐layer approach increases the capacity of depth filters by preventing the filter from fouling too early, it allows larger particles to be captured in the first layer and smaller particles in the second layer. The nominal retention rating decreases with each layer as seen in the SEM images in Figure 2. In the primary D0HC filter, layer 1 is only composed of cellulose fibers with large spaces for the capture of cells and larger debris, while in layer 2 we see the introduction of DE as a filler. DE reduces the pore size of the filter but also provides an increased surface area for the removal of impurities by adsorption. In the secondary X0HC filter, both layers are full of DE making the cellulose fibers difficult to see. The primary filter has a nominal micron rating from 0.6 to 9 μm, while the secondary filter is <0.1 μm.

Diatomaceous earth can vary in size and quality based on where it was mined and how it is subsequently processed. The potential variability in this raw material can add to the lot‐to‐lot variability of the depth filters. In contrast, the Millistak+ HC Pro series has been made from fully synthetic materials in an attempt to address such concerns. The nominal pore rating for the equivalent D0SP and X0SP filters is the same; however, there are some obvious differences that can be seen in the SEM images. The primary D0SP has two additional layers compared to the D0HC, helping to improve solid capacity. The first two layers are made from polyacrylic fibers only with the first layer being a very open non‐woven structure. The silica filler is introduced in the 3rd layer and there is significantly more silica in the last layer compared to DE in the final layers of the D0HC. The X0SP is filled with silica. The size of the silica filter becomes smaller from the D0SP to the X0SP. In the X0SP, the silica is finer and more uniform compared to the DE in X0HC.

A switch from the cellulose to the synthetic‐based filter train would add advantages in terms of solid capacity especially with the help of the additional media layers in the primary filter. However, for this work the focus was on the secondary filters as they remove most DNA and HCP.

### 3.2 Flux and impurity breakthrough

**FIGURE 3:**
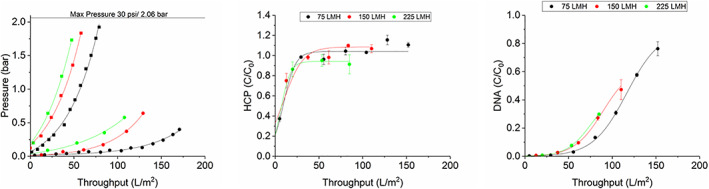
Pressure profile of primary D0HC (▪) and secondary X0HC (•) filters as a factor of volumetric throughput using experimental setup described in Figure 1 at 75 LMH (black), 150 LMH (red) and 225 LMH (green). Experiments were carried out with CHO cell culture with a viability of 57% and a total cell density of 33.6 million cells/ml. DNA and HCP in the feed were 45 ± 0.75 μg/ml and 15.7 ± 0.64 mg/ml, respectively. The IgG titer was 4.2 mg/ml. Panels (b) and (c) show the HCP and DNA breakthrough after the X0HC filter, respectively. Error bar represents 1SD of three repeats of PicoGreen and HCP assays. Throughput was adjusted for the hold‐up volume of the filters

Figure 3 shows the results from filtration experiments carried out on the set‐up described in Figure 1. Three fluxes (75, 150, 250 LMH) were compared and the breakthrough of DNA and HCP was measured after the secondary filter. Figure 3a shows the pressure profile of the three conditions and as expected there is an earlier and sharper pressure increase with higher flux as described by the interception mechanism. At higher flux the inlet side of the filter gets blocked faster, reaching max pressure and the throughput is reduced. Reduced flux is a common method used in manufacturing to increase the solids capacity of the filter. However, there is often a balance between the filter capacity and processing time available in a manufacturing setting.

The HCP concentration was calculated by subtracting the IgG concentration from Total protein concentration, as measured by a BCA assay.^17^ This was found to be a more sensitive method for measuring HCP compared to a CHO HCP ELISA. As the sample at harvest is so crude, the HCP ELISA was not able to give reliable measurements.

There was an almost immediate breakthrough of HCP during the filter loading process and also little overall reduction of HCP. As can be visually observed in Figure 3b, flux did not have an effect on the HCP breakthrough. While previous literature has reported significant HCP reduction with the use of different depth filters, they have used model proteins or cell culture at lower cell density. For example a recent paper used cell culture at 20 million cells/ml and the overall HCP capacity of the depth filters was low.^18^ However, when compared to the group previous work,^19^ they conclude that different depth filter media has different capacities for model protein solutions and some filters may be used to remove specific sub‐classes of HCP. A direct comparison of X0HC and X0SP^20^ showed that X0SP had a 50% higher binding capacity when model protein solutions were used, crediting the improved charge functionality on the X0SP. An increased hydrophobic score was also linked to the reduction of high and low molecular weight mAb species,^21^ though the filter media and filter loading were the biggest factors for this. 3M have shown the ability of Emphaze AEX to remove significant HCP and DNA and to protect ProA resins^15^, ^22^, ^23^, ^24^ however their cell density is below 8 million cells/ml.

While studies with model proteins are invaluable to our understanding of binding mechanisms of HCP, they cannot provide representative information of what happens in the depth filters when using cell culture. Especially when moving to high cell density, the feed material becomes very complex, cells and cell debris, aggregates and colloidal matter, HCP, DNA, spent media, and so forth. Electrostatic charge is the main mechanism by which HCPs are removed and these binding sites are easily available when using model proteins solutions or low cell density feed. However, with all the other components from a high cell density feed, these sites get blocked and are not available for HCP. Therefore to achieve any significant reduction of HCP in the depth filter stage, there is the need for additional filter stages and/or increased filter size.

Though perhaps a small improvement in DNA capture was seen by operating at 75 LMH. As size exclusion affects DNA retention,^25^ it is plausible that there would be a delayed breakthrough at lower flux. The difference in final throughput reached is a factor of the flux, that is, how early the filter reached max pressure.

### 3.3 Filter type, cell culture viability, & DNA breakthrough

As the DNA breakthrough did not reach 100% and the secondary filters also had not reached the max pressure in Figure 3, the next part of the experiments was designed to look at the breakthrough of DNA in more detail. The primary filtration step was increased to a 270 cm^2^ D0HC pod and the intermediate filtrate was collected, which was then passed through either a cellulose‐based (X0HC) or a synthetic‐based (X0SP) 23 cm^2^ pod. The cell culture was harvested on different days to create feed material at varying viability. The details of the cell culture can be found in Table 1. There is a linear relationship (*R* ^2^ = 0.977) between the percentage of lysed cells and DNA concentration in the intermediate pool, indicating most of the free DNA is due to cell lysis. HCP remains relatively constant indicating that most of it is extracellular, perhaps including media components.

**TABLE 1:** Characteristics of cell culture at harvest and impurity concentration (DNA measured by PicoGreen and HCP = Total protein − IgG) in the intermediate pool, used in experiments described by Figure 6. Error bars indicate three repeats of the assay. The %lysed cells/ml was calculated using the following equation: %lysed cells/ml = (1 − %viable cells)*cell density

Cell culture conditions at harvest					Impurity levels in the intermediate pool	
Viability (%)	Total cell count (million cells/ml)	% Lysed cells/ml	Harvest day	IgG titer (mg/ml)	DNA conc. (μg/ml)	HCP (mg/ml)
87	32.4	4.2	9	3.0	6.4 ± 0.28	*6.7 ± 0.32*
67	33.6	11.1	13	6.7	18.5 ± 0.34	*9.4 ± 0.17*
48	33.1	17.2	13	6.0	19.1 ± 0.91	7.7 ± 0.03
37	37.7	23.8	14	6.0	30.2 ± 1.25	9.6 ± 0.39

This work aimed to look at the secondary filters and their ability to remove DNA and HCP, hence the primary filter was not changed. The synthetic equivalent filter, D0SP, has an additional two layers in the pod, which would have altered the composition of the intermediate pool. HCP breakthrough data is not shown as there was no difference seen due to filter type or viability. A cholesterol assay was performed on the breakthrough samples however the data was inconclusive. It is believed this was because the samples were frozen due to the logistics of the project.

The characteristics of the cell culture material are outlined in Table 1, with cell density reaching similar levels for all viabilities. With the change to the synthetic secondary filters, there was an increase in the throughput of 28% for viabilities 37%–67%, while at 87% viability the throughput was 51% higher. The pressure profile was lowest for the highest viability at 87%, which was harvested on day 9. While this gave the best pressure profile and the lowest DNA breakthrough, it is unlikely to be used in a manufacturing setting as the IgG titer was still low, only half compared to harvesting on day 13 or 14.

The outlier in terms of pressure is the condition at 37% viability which performed better than expected. This was the case for both the cellulose and synthetic filters. This was also seen in other work (data not shown here). At present it is not clear what is causing this behavior. One explanation might be that the harvest material is undergoing an aggregation/flocculation behavior at this low viability, which was removed by the primary filter.

The DNA breakthrough curves of cellulose and synthetic filters overlap for each given viability, suggesting there is no change in DNA capture based on filter materials. Significant factors are DNA concentration at the input, which is directly correlated to cell culture viability and harvest day. One important factor of course is the size of DNA, as has been demonstrated in recent literature,^25^ where it was shown that larger, genomic DNA is retained at the top of the depth filter, while smaller DNA fragments are absorbed throughout the depth of the filter.

Publications on the 3M AEX Emphaze filter, based on charged nanofibers,^15^, ^22^, ^23^, ^24^, ^26^ describe the application of this filter as highly effective for the removal of HCP and DNA especially, due to its negatively quaternary amine functionality. However, experiments have been conducted at a cell density <7.7 million cells/ml and the recommend feed turbidity should not be higher than 40NTU. Hence DNA size and the charge of the filters both play a role in the retention of DNA. However based on the data presented in this article these mechanisms become less significant when dealing with complex feed at high cell density and to achieve similar levels of DNA reduction, larger filter areas would be required.

### 3.4 Method development for confocal imaging of depth filters

A novel method was developed for imaging the fouled depth filters. The work mainly focused on the DNA distribution within the filters. Previous methods which had been applied to visualizing resin^27^, ^28^ under the confocal microscope were not applicable as depth filters were too thick for the laser to penetrate the entire depth, which in this case was 4 mm. Literature reports confocal microscopy only being able to visualize at a max. depth of approx. 50–60 μm.^29^ Hence in this method, the filter is cut into thin slices using a cryostat.

The CLSM has been used in literature for the visualization of filters, however they use pre‐tagged proteins, DNA or viral vectors as the feed material^30^, ^31^, ^32^, ^33^ and fouling layers on membranes.^34^, ^35^ While helpful to elucidate binding mechanisms, a dye can affect binding characteristics of the molecule of interest and/or the feed volumes used were significantly lower compared to what the filter would experience in a real clarification stage. In contrast the method developed in this work uses complex cell culture material to understand what happens in a industrially relevant scenario.

PicoGreen and Nile Red were chosen as the fluorescent dyes in these experiments, though it would be possible to add more dyes to image additional components. However as there was no significant HCP removal in the breakthrough experiments, it was decided to omit the third dye for simplicity. PicoGreen is a dye that becomes fluorescent when it intercalates with ds‐DNA. No literature was found to suggest the binding of PicoGreen would be affected by DNA size. Nile Red is a dye that becomes fluorescent when in a hydrophobic environment. It has been used for many other applications, such as micro‐plastic detection, biofuel assays, and so forth.^36^ In this application, Nile Red signal represents lipids, cell debris particulates, and any aggregates. Positive controls were done with DNA, BSA, and oleic acid which confirmed there was no unspecific binding. Negative control was done with clean filters where fluorescence signal was not significant, hence there was no background subtraction in the image analysis.

### 3.5 Image analysis and trends in foulant distribution

**FIGURE 4:**
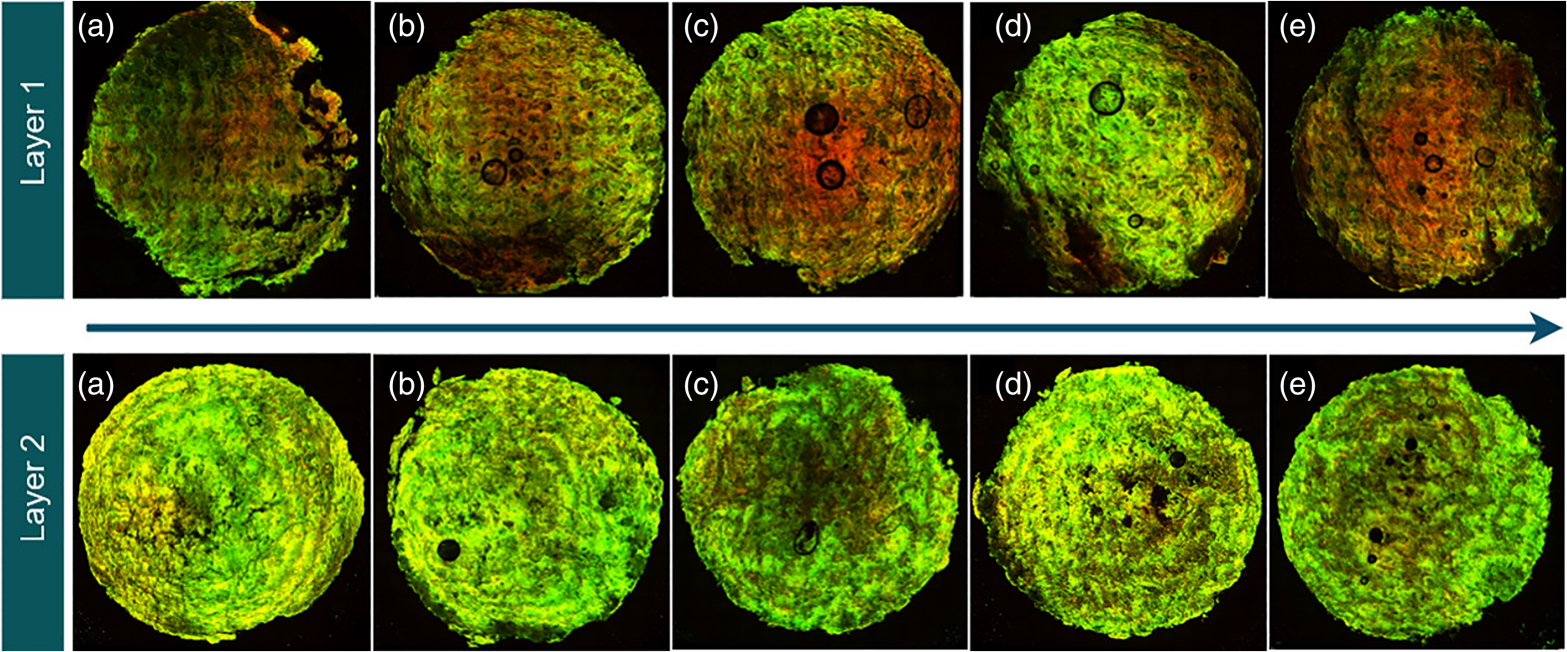
Confocal images of secondary filter X0SP, stained with PicoGreen and Nile Red, after it was used to purify cell culture material at 37% viability. When cutting the filter there was a range of 8–10 slices recovered, where sample (a) is the top and (e) is the bottom of the filter. Samples (b)–(d) were taken from the middle samples. The arrow represents the direction of flow through each layer

**FIGURE 5:**
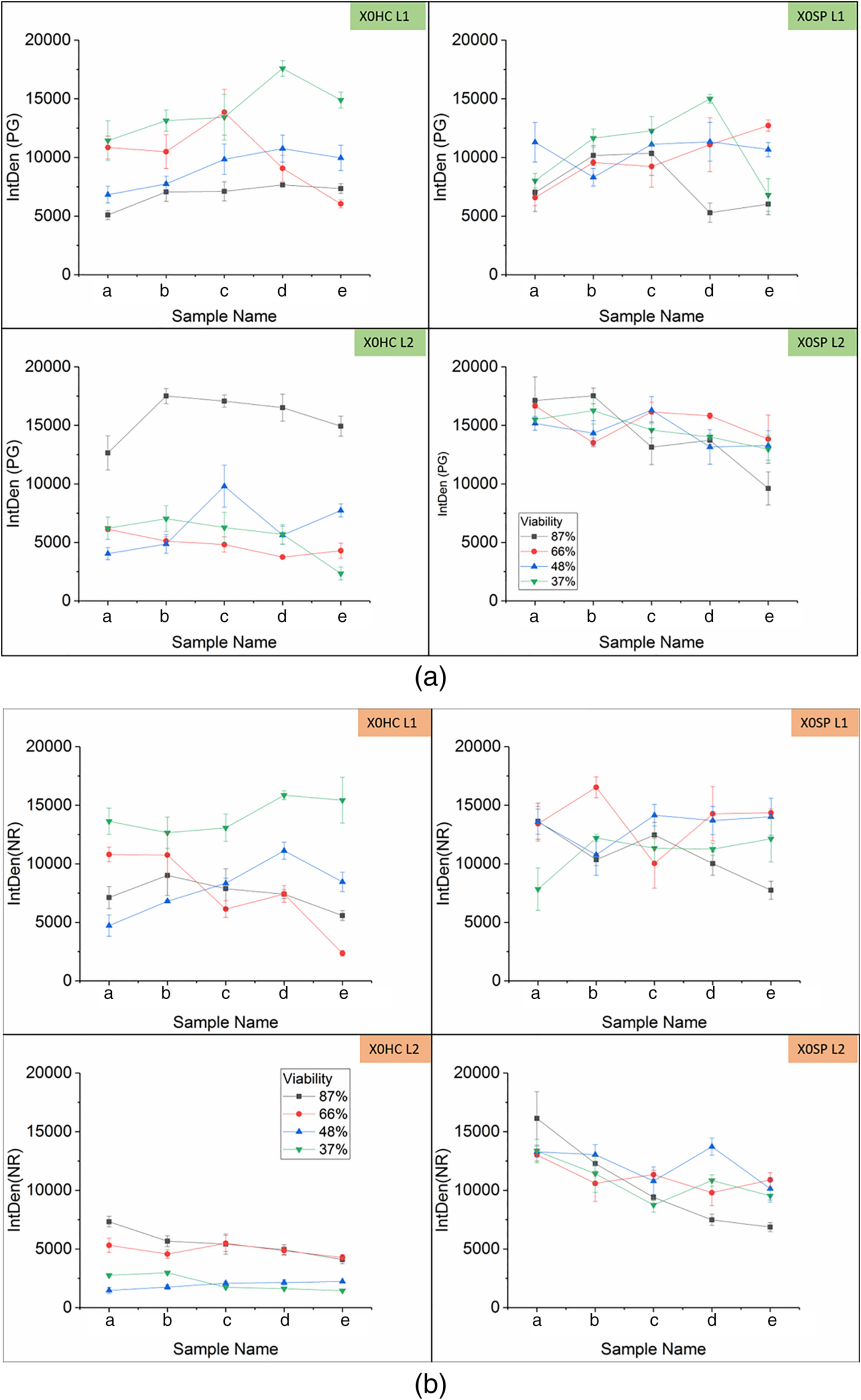
(a) Average integrated density of PicoGreen as a function of filter depth, where the error bar represents 1SD of three measurements. Samples (a)–(e) on the *x*‐axis corresponds to images (a)–(e) in Figure 4. Cell culture viability is indicated by different colors: black 87%, red 66%, blue 48%, and green 37%. (b) Average integrated density of Nile Red as a function of filter depth, where the error bar represents 1SD of three measurements. Samples (a)–(e) on the *x*‐axis corresponds to images (a)–(e) in Figure 4. Cell culture viability is indicated by different colors: black 87%, red 66%, blue 48%, and green 37%

Figure 4 shows the images generated from the secondary synthetic (X0SP) filter after it was used to clarify cell culture at 37% viability. The five images for each layer represent samples taken at different depths from the inlet to the outlet, where images (a)–(e) indicate the samples taken from the top to the bottom of the filter. The differences between Layer 1 (L1) and Layer 2 (L2) can be seen visually. However, the integrated density (IntDen) was measured in ImageJ software, which is defined as the total sum of pixels in an area and was measured for the PicoGreen and the Nile Red individually. This data is shown in Figure 5a,b, where the five points on the *x*‐axis correspond to the five slices from each filter layer. As observed in the images, there are some challenges with the sample preparation method (air bubbles, sample breaking, and brighter signal from the edges of the sample) and as such this data aims to provide trends rather than absolute values.

**FIGURE 6:**
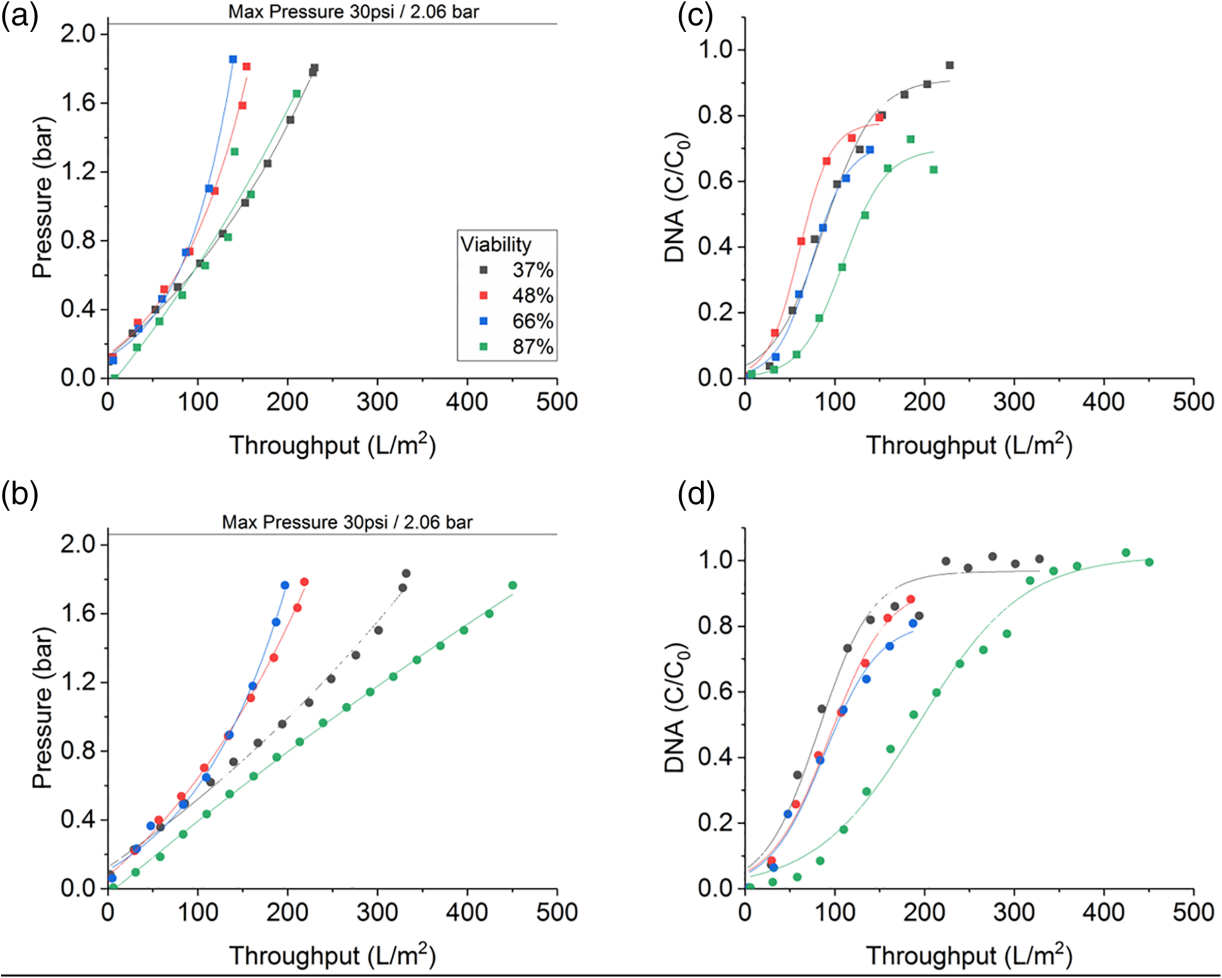
Pressure profile of secondary filters X0HC (a) and X0SP (b) as a function of volumetric throughput, and corresponding DNA breakthrough after secondary X0HC (c) and X0SP (d) using cell culture at different viability described in Table 1. Throughput was adjusted for the hold‐up volume of the filters

In Figure 6, the breakthrough curves of DNA are similar for both filter types. However, with closer inspection under the CLSM, the distribution of DNA in the filters is different. In the X0HC filter, there is higher PicoGreen and Nile Red IntDen in L1 compared to L2, except for sample 87L2 where PicoGreen signal is much higher than expected. One hypothesis is that the fouling build‐up in X0HC is happening in L1, hence we see stronger fluorescent signal there compared to L2, suggesting that the filter is getting blocked in L1 and the additional area of L2 is not being utilized fully. In contrast for X0SP, both layers are being utilized as indicated by higher IntDen in both layers. This could explain the higher throughput in the synthetic filters.

The high PicoGreen IntDen at 87% viability in X0HC L2 may be explained by its early harvest. At this point the cell culture is still in good condition and cells have not started to die yet, hence there has not been an accumulation of debris in the feed material. This means that L1 does not get blocked as quickly and there is less debris to foul the filter, hence L2 is fully utilized for the capture of DNA. The mass of DNA retained on the filter (calculated bases on the input and output concentration) was found not to have any relationship to the Total PicoGreen fluorescence. However these fluorescence measurements are not to be taken as absolute values but rather as an indication of trends when comparing different filters and/or operating conditions.

**FIGURE 7:**
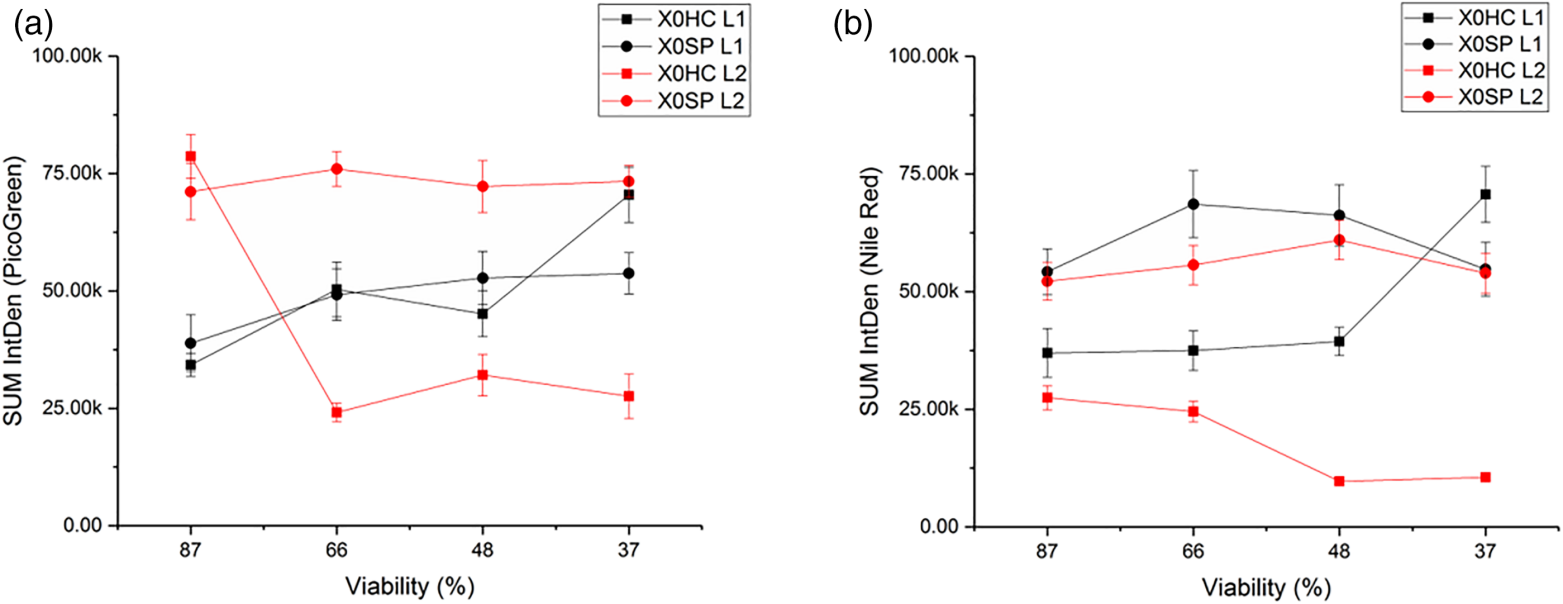
The sum of PicoGreen (a) and Nile Red (b) IntDen as a function of cell culture viability, where black is L1 and red is L2 for X0HC (squares) and X0SP (circles)

The sum of IntDen across each layer is shown in Figure 7 for PicoGreen and Nile Red individually. Again except for the 87% sample, in X0HC the total PicoGreen IntDen is higher in L1 and it increases as cell culture viability drops. The opposite is true of X0SP where the PicoGreen IntDen is higher in the second layer but no changes are seen with viability. However at 37% viability in L1 there is a significant increase in PicoGreen signal. In terms of Nile Red, there is a build‐up in L1 of X0HC where it remains constant for 87%–48% viability, but then we see a jump at 37%. The opposite is the case for L2, where values are lower than in L1 and decrease with lower viability. Whereas X0SP filters have similar levels of Nile Red IntDen across both layers and viabilities. This supports the theory that the X0HC filters are getting blocked in L1 and are not fully utilized. The 30% increase in throughput seen with X0SP filters compared to X0HC filters, also corresponds to an increase of approx. 30% in the Total PicoGreen IntDen (Figure 7), except at 87% viability where the PicoGreen IntDen is similar to the synthetic filter but the throughput is higher.

While viability is important, the harvest day itself may be a factor in the clarification of cell culture, as the 66% and 48% viability gave similar results (harvested on the same day) in terms of pressure, throughput, DNA breakthrough and even PicoGreen and Nile Red IntDen trends. Very early (Day 9 and 87% viability) and very late (Day 14 and 37% viability) are unlikely scenarios for any manufacturing process, however they were chosen as extremes on both ends to see the effect on the depth filtration.

## 4 CONCLUSION

Previous literature has demonstrated the mechanism of DNA and HCP removal during depth filtration by investigating components individually or at cell density <10 million cells/ml. In this work, we have shown that removing process related impurities is very challenging when using CHO cell culture at high cell density. At ~30 million cells/ml the HCP breakthrough is immediate and there is no change in HCP reduction with either viability, filter material or flux. The DNA concentration in the feed increases with lower viability (later harvest day) which affects the retention of DNA in the depth filter. There is a delayed breakthrough when the viability is high. The breakthrough data also agrees with the confocal imaging data where the distribution of foulant in the depth filter changes with filter type and viability.

The complex material containing, cell debris, aggregates, and so forth, takes up the surface area available for binding, resulting in reduced retention of HCP and DNA. This makes it challenging to remove process related impurities without the use of a large filter area. This may prove beneficial, by extending the lifetime of chromatography resin, for example, however a cost–benefit analysis would be required for any given process.

CLSM is an invaluable tool to understand the distribution of the foulant in the different layers. In combination with the breakthrough studies, it gives a better understanding of how complex feed behaves. This is important as it can help inform process development and also aid in the design of new depth filters.

## CONFLICT OF INTEREST

All authors declare that they have no conflicts of interest.

## AUTHOR CONTRIBUTIONS

**Maria Parau:** Conceptualization (lead); data curation (lead); formal analysis (lead); investigation (lead); methodology (lead); project administration (lead); writing – original draft (lead). **Thomas Johnson:** Data curation (supporting); methodology (supporting); writing – review and editing (equal). **James Robert Pullen:** Conceptualization (equal); funding acquisition (equal); resources (equal); supervision (equal); writing – review and editing (equal). **Daniel Gilbert Bracewell:** Conceptualization (equal); funding acquisition (equal); project administration (equal); resources (equal); supervision (equal); writing – review and editing (equal).

### PEER REVIEW

The peer review history for this article is available at https://publons.com/publon/10.1002/btpr.3233.

## Data Availability

The data that support the findings of this study are available from the corresponding author upon reasonable request.
